# A 40 GHz High-Image-Rejection LNA with a Switchable Transformer-Based Notch Filter in 65 nm CMOS

**DOI:** 10.3390/mi16060676

**Published:** 2025-05-31

**Authors:** Yutong Guo, Jincai Wen

**Affiliations:** Key Laboratory of Radio Frequency Circuits and Systems, Ministry of Education, Hangzhou Dianzi University, Hangzhou 310018, China; gyt20000109@163.com

**Keywords:** low-noise amplifier, notch filter, poles and zeros, image rejection

## Abstract

This article presents a low-noise amplifier (LNA) with high image rejection ratio (IRR) operating in the 5G millimeter-wave band using a 65 nm CMOS process. The circuit adopts an inter-stage notch filtering structure composed of a transformer and a switched capacitor array to achieve image suppression and impedance matching with no die area overhead. By adjusting the values of the switch capacitor array, the transmission zeros are positioned in the stopband while the poles are placed in the passband, thereby realizing image rejection. Furthermore, the number and distribution of poles under the both real and complex impedance conditions are analyzed. Moreover, the quality factor (*Q*) of the zero is derived to establish the relationship between *Q* and the image rejection ratio, guiding the optimization of both gain and IRR of the circuit design. Measurement results demonstrate that the LNA exhibits a gain of 18 dB and a noise figure (NF) of 4.4 dB at 40 GHz, with a corresponding IRR of 53.4 dB when the intermediate frequency (IF) is 6 GHz. The circuit demonstrates a 3 dB bandwidth from 36.3 to 40.7 GHz, with an IRR greater than 42 dB across this frequency range. The power consumption is 25.4 mW from a 1 V supply, and the pad-excluded core area of the entire chip is 0.13 mm².

## 1. Introduction

Millimeter wave technology plays a key role in fifth generation (5G) wireless communication, offering high bandwidth, low latency, and high data rates. The 5G millimeter-wave (mmWave) frequency band demonstrates significant system advantages through multi-band coordination, with the main operating frequency ranges including 24.25–27.5 GHz, 26.5–29.5 GHz, 27.5–28.35 GHz, and 37–40 GHz. In millimeter-wave transceiver front-end architectures, the low-noise amplifier (LNA) serves as a core module, and various innovative technical solutions have been developed to optimize its performance. Recent research on millimeter-wave LNA design has achieved remarkable progress. Numerous key technologies have been proposed to simultaneously achieve high gain and low noise figure (NF), significantly enhancing LNA performance [1,2,3,4]. Substantial research outcomes have also been obtained in broadband characteristics and low-power design [5,6]. However, in superheterodyne receivers, the nonlinear characteristics of the mixer cause both the desired signal and its image signal to be down-converted to the intermediate frequency (IF), resulting in signal overlap and interference. Therefore, image rejection techniques must be employed to suppress interference from the image frequency band, thereby improving the signal-to-noise ratio (SNR) of the received signal and ultimately enhancing overall system performance.

Several feasible methods have been proposed to address image interference issues. At the system level, the following approaches are currently available. For example, the transceiver front-end architecture incorporates a filter and implements an RF chopper switch to achieve transmitter self-interference suppression [7]. Alternatively, programmable RC networks can be incorporated into the transceiver system to achieve IF domain harmonic suppression and pre-calibration [8]. An IQ mixer can also be utilized for harmonic suppression to enhance anti-interference capability [9].

In addition to addressing image interference at the system level, incorporating filtering structures into the LNA can also achieve image rejection. In low-frequency circuits, LNAs utilize LC filter networks to enhance image rejection performance [10,11]. Additionally, a tri-coil electromagnetic coupling circuit is implemented to generate pole-zero pairs for further image frequency suppression [12,13]. At high frequencies, the following methods are also employed. For example, embedding filters within the LNA enables image rejection functionality [14,15]. Furthermore, a hybrid magnetic-electric coupling circuit architecture is proposed. This design precisely controls the cancelation effect between magnetic and electric coupling to generate transmission zeros outside the passband, achieving both image rejection and impedance transformation functionality [16]. The inter-stage and output matching networks incorporate switchable tri-coupled transformers and switchable capacitor arrays to achieve high image rejection performance [17]. A frequency-reconfigurable image rejection filter for the 5G FR2 band is proposed, utilizing a pole-zero inversion topology that switches between high-pass and low-pass modes to interchange poles and zeros [18]. However, this work only designs the filter and does not integrate it into a complete circuit.

**Figure 1:**
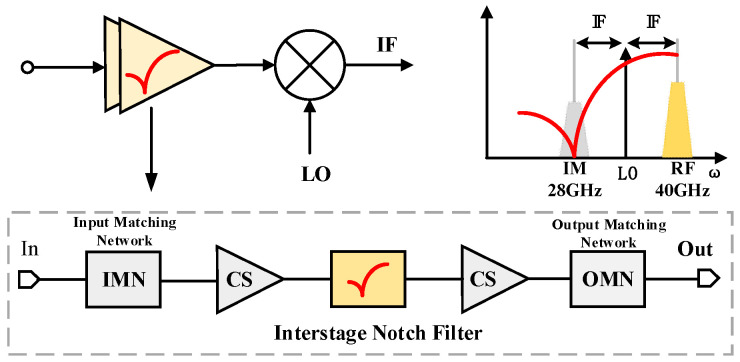
Schematic diagram of image rejection LNA.

For the application scenarios of multi-band millimeter wave receivers, there is a problem of image signal interference between different frequency bands [19,20,21,22,23]. As shown in Figure 1, when the receiver uses a local oscillator (LO) signal source for upper or lower sideband mixing reception, the signals in the 28 GHz and 40 GHz frequency bands will be mirror images of each other, and the corresponding IF will be 6 GHz.

Aiming at the challenges of achieving low noise amplification in the millimeter-wave receiving circuit as well as high image signal suppression, this article realizes 36.3–40.7 GHz LNA based on a 65 nm CMOS technology using a compact notch filter matching structure consisting of a transformer and a switch capacitor array. The organization of this article is as follows: Section 2 focuses on introducing the pole-zero analysis of the notch filter structure and the quality factor (*Q*) at zero and IRR effect; Section 3 presents the design method of the 40 GHz LNA; Section 4 demonstrates the experimental results of the LNA; and Section 5 draws the conclusions.

## 2. Analysis of the Switchable Transformer-Based Notch Filter

**Figure 2:**
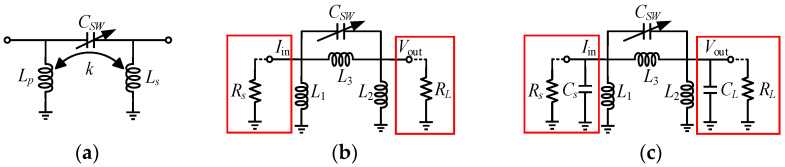
(**a**) Switchable notch-filtering structure; (**b**) π-network model under real impedance conditions; (**c**) π-network model under complex impedance conditions.

The proposed switchable notch filter is shown in Figure 2a. The filtering structure is composed of a transformer and a switchable capacitor. This structure establishes poles and zeros through the transformer and the capacitor. Furthermore, the positions of the poles and zeros can be adjusted using the switchable capacitor *C_SW_*, so as to expand the operating range and achieve signal suppression over broadband frequencies.

When considering the situation of two cases in which the port impedance is classified into real impedance and complex impedance, respectively, the π-network models of the switchable filtering structure are shown in Figure 2b,c, and can be used to analyze its pole-zero characteristics.

### 2.1. Pole-Zero Analysis

First, consider the case in which the port impedance is real impedance, as shown in Figure 2b. At this time, the notch filter can be used as an independent filtering circuit. The transformer structure can be modeled as a π-type network, in which each inductor can be expressed as follows, where *L_p_* and *L_s_* are the primary and secondary coils of the inter-stage transformer, *k* is the transformer coupling coefficient, *C_SW_* is the switch capacitance, and *L_m_* is the mutual inductance between transformer windings, describing their magnetic coupling intensity:

(1) $$L_{1}=\frac{L_{p}L_{s}-L_{m}^{2}}{L_{s}-L_{m}},\;L_{2}=\frac{L_{p}L_{s}-L_{m}^{2}}{L_{p}-L_{m}},\;L_{3}=\frac{\left(1-k^{2}\right)\sqrt{L_{p}L_{s}}}{k}$$

In this design, *Z*_21_ is used in simulations to clearly show the effect of poles and zeros on gain, while *S*_21_ is used in measurements to match standard RF testing. For differential circuits with impedance matching, *Z*_21_ and *S*_21_ are related, and *Z*_21_ trends qualitatively reflect *S*_21_ responses. Therefore, *Z*_21_ is used to obtain the poles and zeros of the circuit. The transfer impedance of the switchable notch filter, *Z*_21_, can be derived as

(2) $$Z_{21}=\frac{s\sqrt{L_{p}L_{s}}\left[k+s^{2}C_{SW}\sqrt{L_{p}L_{s}}\left(1-k^{2}\right)\right]}{1+s^{2}C_{SW}\left(L_{p}+L_{s}-2k\sqrt{L_{p}L_{s}}\right)}$$

Accordingly, the transmission pole *ω_p_* and transmission zero *ω_z_* are as follows:

(3) $$\omega_{p}=\frac{1}{\sqrt{C_{SW}\left(L_{p}+L_{s}-2k\sqrt{L_{p}L_{s}}\right)}}$$

(4) $$\omega_{z}=\sqrt{\frac{1}{C_{SW}L_{3}}}$$

**Figure 3:**
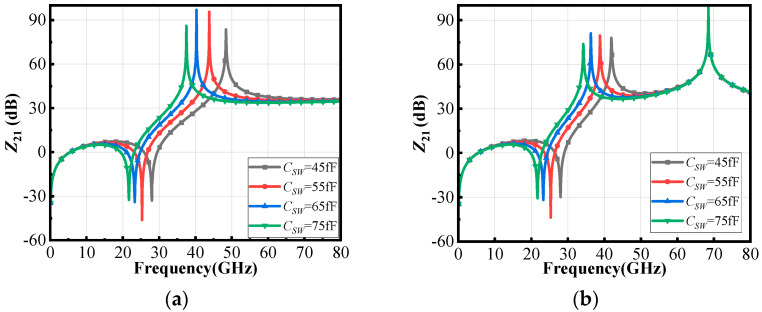
The pole-zero characteristics of the notch filter and its relationship with *C_SW_* under (**a**) real impedance and (**b**) complex impedance.

It can be seen that when both the source impedance and load impedance are pure real impedance, this switchable notch filter structure has only one pole and one zero, as shown in Figure 3a. Furthermore, the pole-to-zero frequency ratio is

(5) $$\frac{\omega_{p}}{\omega_{z}}=\sqrt{\frac{\left(1-k^{2}\right)\sqrt{L_{p}L_{s}}}{k\left(L_{p}+L_{s}-2k\sqrt{L_{p}L_{s}}\right)}}$$

It can be found from (5) that the ratio of pole to zero only depends on the transformer parameters and is independent of *C_SW_*. Therefore, by adjusting the switching capacitor value *C_SW_*, it is possible to achieve a proportional migration of the poles and zeros, enabling pole-zero tracking.

When the switchable notch filter is integrated into a circuit, for example, embedded in an LNA as the inter-stage matching network to suppress the image signal, the impedances of its two ports are complex impedances. Under such circumstances, the port impedance can be expressed as the equivalent resistors (*R_S_*, *R_L_*) and capacitors (*C_s_*, *C_L_*) of the preceding and succeeding stage amplifiers, as shown in Figure 2c. The *Z*_21_, pole expressions, and the ratio of pole to zero of the circuit can be expressed as follows:

(6) $$Z_{21}=-\frac{C_{SW}L_{1}L_{2}L_{3}s^{3}+L_{1}L_{2}s}{As^{4}+Bs^{2}+L_{3}+L_{2}+L_{1}}$$

(7) $$\omega_{p1}=\sqrt{\frac{C-\sqrt{D+E^{2}}}{2F}},\;\omega_{p2}=\sqrt{\frac{C+\sqrt{D+E^{2}}}{2F}}$$

(8) $$\frac{\omega_{p}}{\omega_{z}}=\sqrt{\frac{C_{SW}\left(C-\sqrt{D+E^{2}}\right)\left(\sqrt{L_{p}L_{s}}-kL_{m}\right)}{2Fk}}$$

(9) $$A=L_{1}L_{2}L_{3}\left(C_{L}\left(C_{s}+C_{SW}\right)+C_{SW}C_{s}\right)$$

(10) $$B=L_{1}\left(\left(C_{s}+C_{SW}\right)L_{3}+\left(C_{s}+C_{L}\right)L_{2}\right)+L_{2}L_{3}\left(C_{L}+C_{SW}\right)$$

(11) $$C=L_{1}\left(C_{s}L_{2}+C_{SW}L_{3}+C_{s}L_{3}+C_{L}L_{2}\right)+L_{2}L_{3}\left(C_{L}+C_{SW}\right)$$

(12) $$D=-4L_{1}L_{2}L_{3}\left(L_{1}+L_{2}+L_{3}\right)\left(C_{L}C_{s}+C_{SW}\left(C_{L}+C_{s}\right)\right)$$

(13) $$E=C_{SW}\left(L_{1}+L_{2}\right)L_{3}+C_{L}L_{2}\left(L_{1}+L_{3}\right)+C_{s}L_{1}\left(L_{2}+L_{3}\right)$$

(14) $$F=L_{1}L_{2}L_{3}\left(C_{L}C_{s}+C_{SW}\left(C_{L}+C_{s}\right)\right)$$

Based on the poles and zeros derived from the complex impedance *Z*_21_, one can deduce that there is a zero point in the complex impedance domain which coincides with the one in the real impedance domain. The difference, however, is that owing to the introduction of *C_s_* or *C_L_*, they resonate with *L*_1_ and *L*_2_, respectively, producing two poles. Moreover, under complex impedance conditions, when the *C_SW_* changes, the second pole *ω_p_*_2_ remains nearly unchanged, while only the first pole *ω_p_*_1_ and the zero *ω_z_* change accordingly, as shown in Figure 3b.

**Figure 4:**
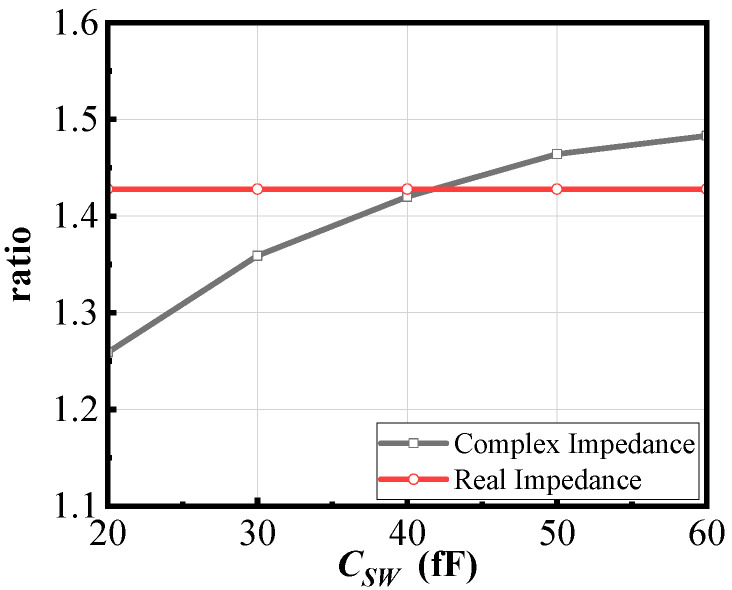
The pole-to-zero ratio characteristics with the capacitor *C_SW_* under two different port impedance conditions.

Furthermore, in the case of complex impedance, the pole to zero ratio depends not only on *k* and the impedance capacitors on both sides, but also on *C_SW_*, meaning it is no longer independent of the switching capacitance. Figure 4 compares the ratio of poles to zeros (ratio) under two different port impedance conditions when the operating frequency and image frequency are 40 GHz and 28 GHz, respectively. It can be observed that, under complex impedance conditions, the pole to zero ratio slightly increases with the increase in *C_SW_*, approximately maintaining a positive correlation.

### 2.2. Quality Factor at Zero and IRR of the Notch Filter

**Figure 5:**
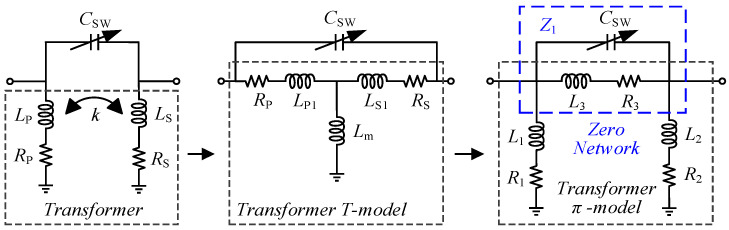
Equivalent T-model and π-model of the notch filter network.

A notch filter can be used to implement an image rejection filter. That is, the zero point can be kept within the stopband and the pole within the passband to achieve the image rejection effect. At this point, it is necessary to minimize the loss at the poles and ensure that the notch depth at the zero is large enough to achieve the maximum IRR. The notch depth of the circuit is determined by the quality factor *Q*, so it is necessary to find the parameters that affect *Q* to ensure that the actual circuit exhibits good performance. When considering the parasitic resistance of notch filters, the *Q* value at the zero determines the overall image rejection of the structure. As shown in Figure 5, the transformer structure is first modeled as a T-model network. Taking into account the parasitic resistance of the inductors, the T-model structure is further transformed into a π-model structure to obtain the zero point network *Z*_1_ of the notch filter. Consider the zero point network, which consists of a parallel resonant network consisting of the capacitor *C_SW_* and the inductor *L*_3_. When *C_SW_* and *L*_3_ resonate, the impedance is very high, and the signal is blocked from input to output, resulting in a zero point.

As shown in Figure 5, the *Q* of the zero network (*Q_z_*) can be determined as follows, where *R*_3_ is the parasitic resistance of *L*_3_.

(15) Qz=ImZ1ReZ1=ωzL3R3

*L_p_*_1_ and *L_s_*_1_ are the inductors in the T-network model, and *R_p_* and *R_s_* are the parasitic resistances of *L_p_*_1_ and *L_s_*_1_. The relationship between *R*_3_ of the π network and the parasitic resistance of the transformer is

(16) $$R_{3}=\frac{R_{p}}{2}\left(1+\frac{L_{s1}}{L_{m}}\right)+\frac{R_{s}}{2}\left(1+\frac{L_{p1}}{L_{m}}\right)$$

We can further derive the relationship of *Q_z_* as shown in Equation (17).

(17) $$Q_{z}=\frac{2\left(1-k^{2}\right)L_{p}L_{s}\omega_{z}}{L_{s}R_{p}+L_{p}R_{s}}$$

Let the quality factors of the transformer coils *L_p_* and *L_s_* be *Q_p_* and *Q_s_*, respectively.

(18) $$Q_{p}=\frac{\omega L_{p}}{R_{p}},\;Q_{s}=\frac{\omega L_{s}}{R_{s}}$$

Substitute *Q_p_* and *Q_s_* into *Q_z_* to obtain the relationship between *Q_z_* and *Q_p_*, *Q_s_*, as shown in Equation (19).

(19) $$\frac{1}{Q_{z}}=\frac{1}{2\left(1-k^{2}\right)}\left(\frac{1}{Q_{p}}+\frac{1}{Q_{s}}\right)\approx \frac{1}{2}\left(\frac{1}{Q_{p}}+\frac{1}{Q_{s}}\right)$$

**Figure 6:**
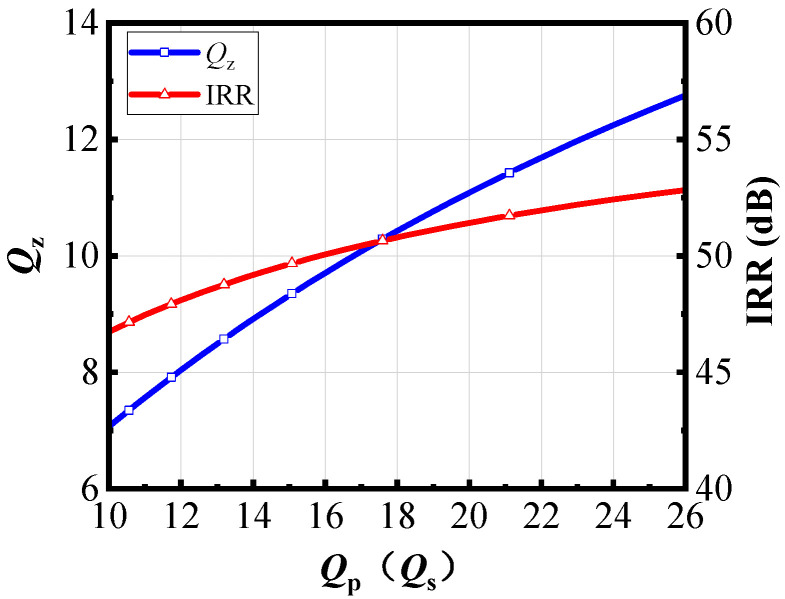
The relationship between *Q_z_*, IRR and *Q_p_*(*Q_s_*).

Figure 6 shows the relationship between IRR, *Q_z_*, and *Q_p_*, *Q_s_* when the notch filter has been designed for a 40 GHz image rejection filter with the transmission zero located at 28 GHz. It can be observed that IRR and *Q_z_* are directly proportional to *Q_p_* and *Q_s_*. During the design process, the maximum *Q_p_* and *Q_s_* can be selected to achieve the maximum *Q_z_*. Since the IRR is primarily related to the quality factor at the zero point, a higher *Q_z_* at the zero point results in greater IRR. When determining the structure of the transformer, it is necessary to maximize its quality factors *Q_p_* and *Q_s_* as much as possible and minimize the losses of the transformer in the design.

## 3. The 40 GHz LNA Circuit Design

### 3.1. Overall Circuit Design

**Figure 7:**
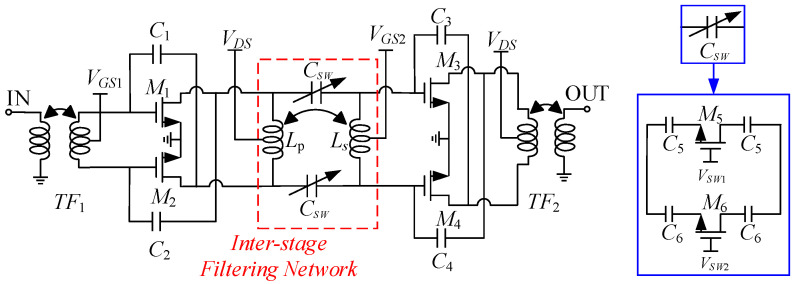
Schematic diagram of the overall LNA circuit.

A high-image-rejection LNA circuit with an operating frequency of 40 GHz was designed using 65 nm CMOS technology, as shown in Figure 7. The circuit consists of two stages of differential neutralized common-source structures, with *M*_1_, *M*_2_, *M*_3_, and *M*_4_ as differential common-source transistors. *M*_5_ and *M*_6_ act as switches for the switched capacitor array, while *C*_5_ and *C*_6_ are fixed capacitors that help adjust the switched capacitance. Capacitors *C*_1_, *C*_2_, *C*_3_, and *C*_4_ serve as neutralization capacitors. Transformers *TF*_1_ and *TF*_2_ provide input/output matching. Inductors *L_p_* and *L_s_*, together with *C_SW_*, form the inter-stage notch filter. By introducing neutralization capacitors (*C*_1_, *C*_2_, *C*_3_, *C*_4_) to establish negative feedback paths, the gate-to-drain capacitance (*C*_gd_) effects of the transistors are eliminated, thereby achieving improved gain and enhanced stability. The input and output matching network is composed of a pair of transformers for impedance matching. The inter-stage matching structure consists of a transformer and an array of two sets of switching capacitors, which together form a notch filter structure that achieves both inter-stage matching and out-of-band rejection. *V_GS_*_1_ and *V_GS_*_2_ are the gate voltages, *V_DS_* is the drain voltage, and *V_SW_*_1_ and *V_SW_*_2_ are the switches of the switched capacitor array.

**Figure 8:**
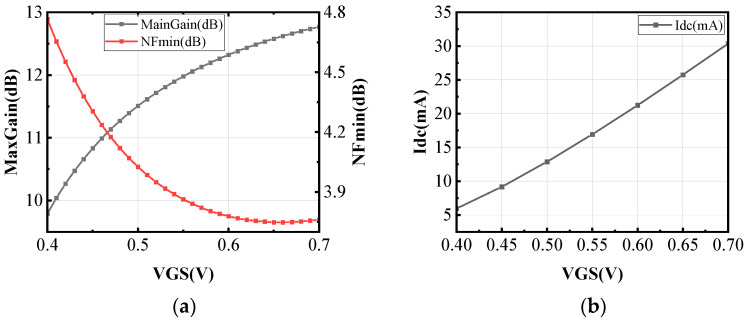
The impact of (**a**) gate voltage on gain and noise and (**b**) gate voltage on power consumption.

DC bias selection is crucial in circuit design. For maximum gain, drain voltage is set to the transistor’s 1 V safety limit. The gate voltage must optimize both the gain and noise figure in the differential common-source configuration. Figure 8 shows the simulated variation in maximum gain (MaxGain) and minimum noise figure (NFmin) of the differential common-source structure with respect to the gate voltage *V_GS_*, while also considering the impact of gate-source voltage *V_GS_* on power consumption.

As the gate voltage increases, the gain gradually rises and approaches a stable value, while the noise figure gradually decreases and then levels off. Meanwhile, power consumption increases with higher gate voltage. To meet gain and noise requirements under low-power conditions, the transistor’s maximum current handling capability must also be considered. In this case, a gate voltage of 0.5 V is chosen. Under the 65 nm CMOS process, the safe drain voltage for transistor testing is 1 V; therefore, a drain voltage of 1 V is selected in this work, and in the actual design, the bias lines are orthogonal to the transformer.

### 3.2. Switched Capacitor Array

The notch filter matching structure is a key part of circuit design, and by embedding it in the inter-stage of the amplifier, it can minimize the impact on the circuit gain and noise figure as much as possible. Based on the analysis in Section 2, the design process of the notch filter matching structure in this circuit is as follows:

Step 1: Considering that the impedance capacitors at both ends do not differ significantly, start by assuming that *L_p_* = *L*_s_. Using Equations (3)–(5), the parameters *L_p_*_,_ *L_s_*, *k*, and *C_SW_* under real impedance conditions can be determined. For example, when *ω*_p1_ = 40 GHz, *ω*_z_ = 28 GHz, the values of *L_p_*, *L_s_*, *k*, and *C_SW_* are obtained. These values are applicable to a single-ended circuit; in actual differential applications, the inductance values need to be multiplied by two.

Step 2: Based on Figure 4, under the same *k*, the pole-to-zero ratio for complex impedance is smaller than that for real impedance. Using the parameters *L_p_*, *L_s_*, *k*, and *C_SW_* values obtained from Step 1 and substituting them into the complex impedance equations shows that the current pole-to-zero ratio will be reduced. To restore the pole-to-zero ratio to the desired value, *k* needs to be decreased to increase the pole-to-zero ratio.

Step 3: Adjust both *k* and *C_SW_* for the complex impedance. While decreasing *k*, simultaneously adjust *C_SW_* to move the zeros and poles to achieve the desired values.

Step 4: Based on the relationship of *Q*_z_ and the process characteristics, determine the physical dimensions of the transformer structure and capacitors.

**Figure 9:**
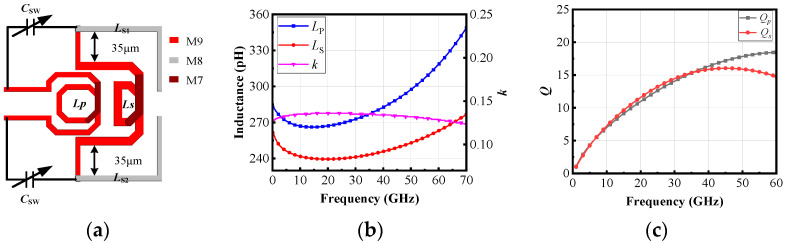
(**a**) The layout of the transformer, and (**b**) the inductance of *L_p_* and *L_s_* and their coupling factor, and (**c**) the *Q_p_* and *Q_s_* of *L_p_* and *L_s_.*

Since the zero frequency of the inter-stage filtering structure needs to be set to 28 GHz, according to (1) and (4), it can be determined that the coupling coefficient *k* of the transformer satisfies *k* > 0, and the alternating current in the primary coil of the transformer is in the same direction. Therefore, the structure of the inter-stage transformer can be determined, as shown in Figure 9a. The coils *L_p_* and *L_s_* are designed with current in the same direction, and two additional inductors, *L_s_*_1_ and *L_s_*_2_, are introduced. Since the coils *L_p_* and *L_s_* do not overlap, both coils can be designed using the topmost thick metal layer (M9) to reduce losses. When connecting *L_s_*_1_ and *L_s_*_2_, the magnetic coupling between these inductors and *L_s_* must be considered. Although this coupling coefficient is small, it affects the overall coupling coefficient of the transformer. Thus, *L_s_*_1_ and *L_s_*_2_ connections use another metal layer (M8), which has weaker coupling with the upper M9 layer, minimizing the impact on the overall coupling coefficient. The primary coil *L_p_* of the inter-stage transformer has an inductance of 280 pH, while the secondary coil *L_s_* has an inductance of 250 pH. The coupling coefficient between the primary and secondary coils is 0.13. The inductance values of *L_s_*_1_ and *L_s_*_2_ are 120 pH each, as shown in Figure 9b. Figure 9c shows the *Q_p_* and *Q_s_* of the actual transformer. As the frequency increases, the *Q*-factor first rises and then decreases, reaching above 16 for both *Q_p_* and *Q_s_* at 40 GHz.

**Figure 10:**
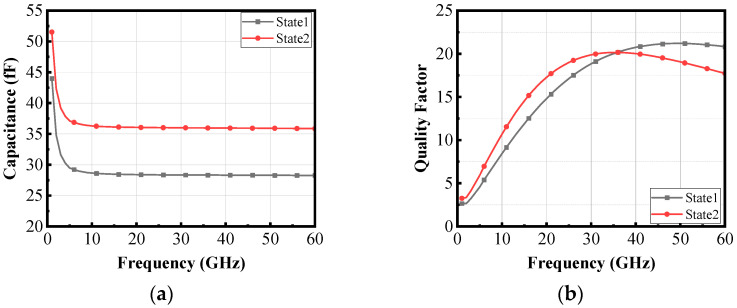
(**a**) The capacitance and (**b**) the *Q*-factor of the switched capacitor array.

The switched capacitor array consists of two parallel sections, each comprising a switched MOS transistor and two fixed capacitors. The switching action (ON/OFF) is controlled by the switched MOS gate voltage *V*_sw_. When the gate voltage is at logic 1, the switch closes, equivalent to a resistor; at logic 0, it opens, equivalent to a capacitor. *V_SW_*_1_ and *V_SW_*_2_ represent the gate control voltages for each bit in the switching array, creating four possible states: 00, 01, 10, and 11. In this design, the 00 and 01 states do not provide optimal gain within the operational frequency band; therefore, only the 10 (State 1) and 11 (State 2) states are implemented in the final design. Considering the actual process, the larger the gate width of each switched MOS transistor, the higher its equivalent capacitance *Q* value in the ON state, and the smaller its equivalent resistance in the off state. Therefore, we chose switched MOS transistors with larger gate widths. For the design of fixed capacitors, we maximize the *Q* value of the capacitor by selecting appropriate metal layers and aspect ratios. By implementing the low loss matching design mentioned above, a high gain of the LNA circuit can be achieved. By selecting the appropriate capacitance value, specifically, when *C_SW_* is in the range of 27–36 fF, the circuit can achieve an operating range covering 37–43 GHz. Therefore, *C_SW_* values within this range were selected for the switched capacitor array. The capacitance value and *Q*-factor of the switched capacitor arrays are shown in Figure 10.

In Figure 3, the *C_SW_* was designed based on the gate-drain impedance of the standalone differential common-source transistor. In Figure 10, the design also considers the effects of input/output matching networks, which alter the gate-drain impedance and adjust the *C_SW_* capacitance accordingly. Additionally, at the 40 GHz operating frequency, the transformer’s inductance and coupling coefficient vary with frequency, causing differences between practical parameters and theoretical calculations, further affecting the *C_SW_* value.

## 4. Measurement Results

**Figure 11:**
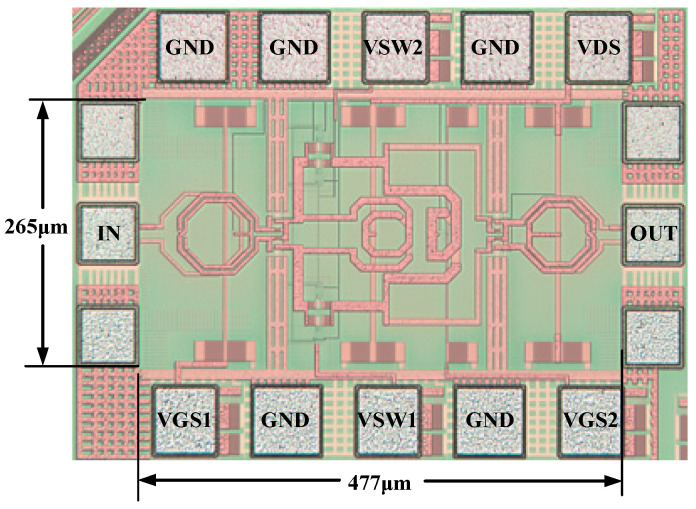
High-image-rejection LNA chip photo.

Figure 11 shows the micrograph of the proposed LNA circuit with a core area of 0.13 mm^2^ and a consumption of 25.4 mW at a 1 V supply.

**Figure 12:**
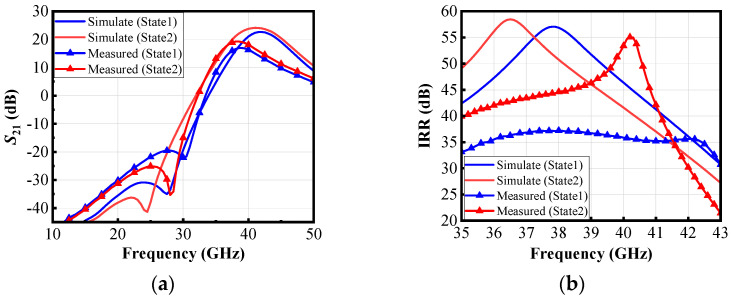
Measured and simulated (**a**) *S*_21_, (**b**) IRR.

**Figure 13:**
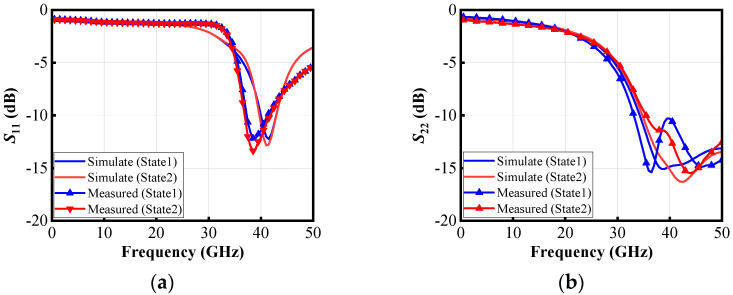
Measured and simulated (**a**) *S*_11_, (**b**) *S*_22_.

As shown in Figure 12a, when the switching capacitor array is adjusted, the measured gain (*S*_21_) reaches 16.3 dB at 40 GHz in State 1 and 18 dB in State 2. The circuit exhibits a 3 dB bandwidth of 36.3–40.7 GHz. As shown in Figure 12b, the measured IRR is 35.6 dB at 40 GHz in State 1 and improves to 53.4 dB in State 2 when the IF signal frequency is 6 GHz, meaning that the LNA circuit features high image-rejection capability. In the frequency range of 35–41.2 GHz, the IRR is consistently greater than 40 dB, while within 32–42 GHz, the IRR remains above 30 dB overall. As shown in Figure 13, at 40 GHz, the input reflection coefficient (*S*_11_) is less than −10 dB in both switching states. Within the 37–42 GHz range, the *S*_11_ remains below −10 dB across the entire band. The output reflection coefficient (*S*_22_) is less than −15 dB in both switching states and the *S*_22_ remains below −10 dB across the range of 33–50 GHz.

Compared to the simulation results, the measurements show a shift toward lower frequencies, primarily due to the accuracy issues of the MOS transistor model and the failure to account for the impact of MOS transistor switching on the transformer.

**Figure 14:**
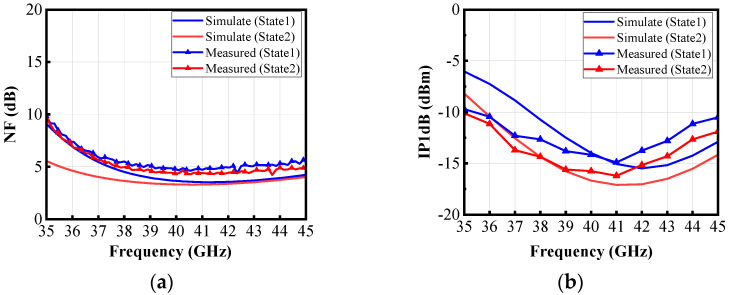
Measured and simulated (**a**) NF, (**b**) IP1dB.

As shown in Figure 14a, at 40 GHz, the NF is 4.7 dB in State 1 and 4.4 dB in State 2, achieving good noise performance. As shown in Figure 14b, in State 1, the test result of input power at 1 dB compression point (IP1dB) in the 3 dB bandwidth is −14.9~−12.3 dBm, and in State 2, the test result of IP1dB in the 3 dB bandwidth is −16.2~−13.7 dBm. The linearity is satisfactory, and the results at 40 GHz are slightly higher than the simulation results because the gain values are lower than the results in the simulation.

**Figure 15:**
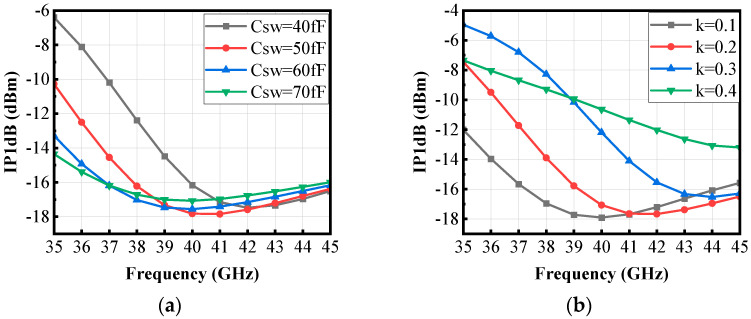
Effects of (**a**) *C_SW_* and (**b**) *k* on IP1dB.

To further analyze the factors affecting circuit performance, the impact of pole positions on linearity (such as IP1dB) is also investigated in this paper. As shown in Figure 15a, increasing the switching capacitor *C_SW_* widens the spacing between the poles, leading to a broader bandwidth but a lower IP1dB, indicating reduced linearity. Conversely, decreasing *C_SW_* narrows the bandwidth and increases IP1dB, thus improving linearity. Figure 15b illustrates that as the coupling coefficient *k* increases, the bandwidth decreases while IP1dB increases, resulting in enhanced linearity. When *k* is reduced, the bandwidth becomes wider, but IP1dB decreases, leading to poorer linearity. This is because in wideband circuits, gain variation across different frequencies is more pronounced. Certain frequencies may experience excessive amplification, pushing the circuit into compression and degrading linearity. Therefore, *C_SW_* and *k* should be carefully optimized based on the target frequency to balance bandwidth and linearity, ensuring the LNA meets performance requirements.

**Figure 16:**
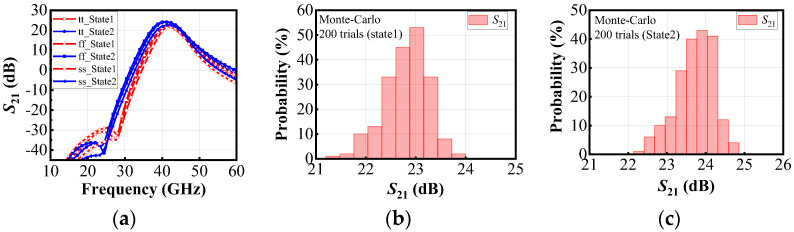
(**a**) Simulation results under different process corners, (**b**) Monte-Carlo simulation in State 1, (**c**) Monte-Carlo simulation in State 2.

To evaluate the robustness and reliability of the design, and to address the frequency discrepancies between measurements and simulations, transistor-level Monte Carlo simulations were further conducted based on this foundation. Figure 16a shows the *S*_21_ simulation results under three process corners: tt, ff, and ss. The results indicate that the gain curves under the three process corners exhibit only minor deviations. Figure 16b and Figure 16c show the Monte-Carlo simulation results under State 1 and State 2, respectively, with 200 sample points generated for each.

Although the simulation results under the three process corners show minor variations, discrepancies with measurement results may still exist. The current Monte Carlo simulations only consider parameter variations in transistors and do not include process and environmental variations in passive components such as capacitors and inductors. Therefore, the simulation results may deviate from actual measurements. Passive components have a significant impact on high-frequency circuit performance, as they can influence parasitic parameters, quality factor (*Q*), and capacitance/inductance values, thereby affecting key metrics such as gain, bandwidth, and linearity. Future work should incorporate parameter variations in passive components and environmental effects into the simulations to achieve more accurate and comprehensive Monte Carlo analyses.

Table 1 shows the performance of the proposed LNAs and compares them to the state-of-the-art results, taking into account gain, NF, and IRR, as well as area, and the proposed high-image-rejection LNA based on inter-stage filtering structures maintains competitive overall performance in the above aspects.

## 5. Conclusions

This article presents a 40 GHz high-image-rejection low-noise amplifier implemented in 65 nm CMOS technology. To achieve superior image rejection, a switchable inter-stage filtering structure was developed. By strategically placing transmission zeros in the stopband and poles in the passband within this filtering structure, effective image rejection is realized. Notably, this architecture simultaneously performs impedance transformation while maintaining its filtering function. Measurement results demonstrate a gain of 18 dB with an NF of 4.4 dB at 40 GHz, while achieving outstanding image rejection of 53.4 dB at the target frequency. This design demonstrates significant advantages in image rejection and chip area, while operating at higher frequencies, making it particularly suitable for 5G millimeter-wave band circuit applications.

## Figures and Tables

**Table 1:** Comparison of image rejection LNA performance.

Ref.	Technology	Freq/GHz	Peak Gain/dB	NF/dB	IRR/dB	Power/mW	Area/mm^2^	IP1dB/dBm
[3]	22-nm FD-SOI	19–36	21.5	1.7–2.2	6.5	17.3	0.05	−24.4~−23.4
		20–36	17.9	2.1–2.9		5.6		
[4]	45-nm RF-SOI	27–46	21.2	2.74–3.24	21.2	25.5	0.38	−21
[13]	22-nm FD-SOI	21.6–34.2	32	2.3	>30	35	0.74	−37.2~−26.6
		21.5–26.5	32.4	2.7				
		27.5–33	33.4	2.3				
[14]	22-nm FD-SOI	20–44	28.5	3.3–5	>75	20.5	1.8 *	−29.5~−25
[15]	0.25-μm SiGe	29–37	28.5	3.1–4.1	>30	80	0.21 *	−22.1
[16]	28-nm CMOS	24–35	22	2.4–3.6	>25	26.8	0.13 *	−18.25
[17]	28-nm CMOS	23.8–33.5	18.1	2.5–3.5	>32.6	14	0.09 *	−19~−15.1
		34.4–41.4	18.9	2.8–3.5				−18.5~−15.2
This work	65-nm CMOS	36.3–40.7	18	4.4–4.9	40–53.4	25.4	0.13 *	−16.2

*: Excluding test pads.

## Data Availability

Data are contained within the article.
